# Injection-Site Sarcoma in a Dog: Clinical and Pathological Findings

**DOI:** 10.1155/2017/6952634

**Published:** 2017-05-09

**Authors:** Terry M. Jacobs, Cathy E. Poehlmann, Matti Kiupel

**Affiliations:** ^1^Park Pet Hospital, 7378 N. Teutonia Ave., Milwaukee, WI 53209, USA; ^2^Department of Pathobiology and Diagnostic Investigation, Michigan State University, 4125 Beaumont Road, Room 152A, Lansing, MI 48910, USA

## Abstract

This case report documents the clinical and pathological findings in a dog that rapidly developed a high-grade sarcoma at the site of multiple vaccinations and follows the response to surgery and adjunct treatment with toceranib. An 11-year-old female spayed Labrador Retriever presented with dorsocervical subcutaneous masses at the injection site three weeks after receiving DA_2_PP-Lepto, Rabies, and Bordetella vaccinations. A high-grade soft tissue sarcoma was diagnosed microscopically and immunohistochemistry revealed positive expression of VEGFr, PDGFr, SCF, and EGFR. Repeat surgical resections and targeted treatment with toceranib resulted in a stable remission for nearly two years.

## 1. Introduction

Injection-site sarcomas commonly occur in cats but are rare in other species [1, 2]. Most injection-site sarcomas occur at the sites of prior vaccinations; however, phenotypically similar sarcomas have been previously reported at sites of injectable medications [3, 4], microchips [5, 6], and implantable devices [7]. Despite considerable research, the pathogenesis of these aggressive tumors and the optimal treatment approach remain uncertain [8]. This case report describes the clinical, pathological, and immunohistochemical findings in a dog with a high-grade soft tissue sarcoma that occurred at a recent vaccination site and the clinical results of surgical resection followed by targeted therapy with toceranib.

## 2. Case Description

**Figure 1:**
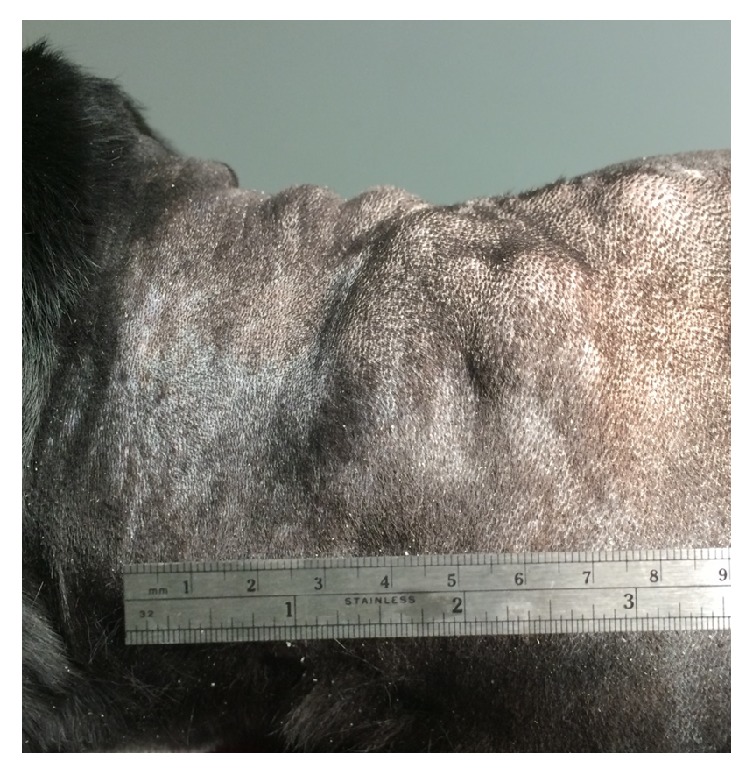
Dorsocervical subcutaneous masses in a dog who received three vaccinations at this site three weeks previously.

An eleven-year-old female spayed Labrador Retriever was presented for evaluation of multinodular subcutaneous masses in the dorsal cervical area (Figure 1).

**Figure 2:**
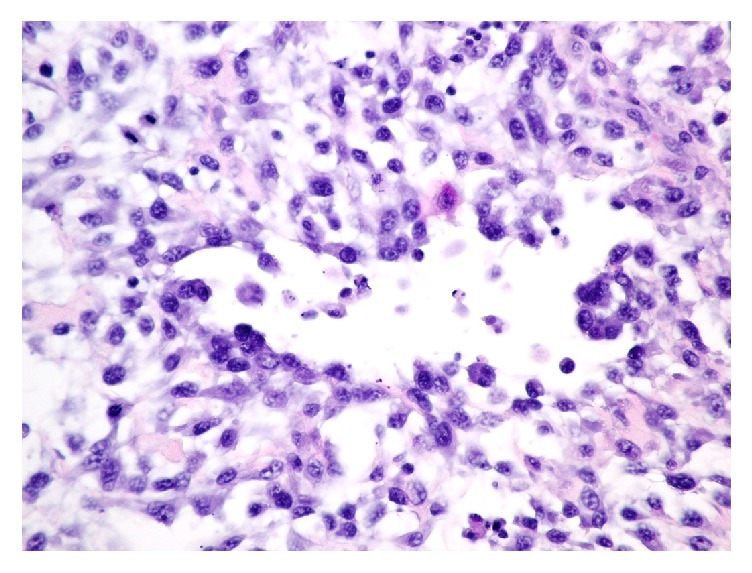
Photomicrograph of a soft tissue sarcoma at an injection site in a dog. Hematoxylin and eosin stain.

Three nonadjuvant vaccinations, DA_2_PP-Lepto, Rabies, and Bordetella, had been injected into this same area during a wellness examination three weeks earlier. The dog's previous vaccination history included routine boosters at standard intervals administered at variable sites since puppyhood. Fine needle aspiration cytology of the masses revealed a mesenchymal spindle cell proliferation with a high level of atypia and minimal numbers of inflammatory cells. A wide surgical excision of the entire site with 3 cm margins was performed the following week. The excised tissue contained a regionally extensive, expansile, and infiltrative spindle cell neoplasm surrounded by a pseudocapsule and mild to moderate chronic inflammation with multifocal lymphonodular aggregates. The center of the neoplasm had undergone extensive necrosis, and the myxomatous matrix was admixed with grey-brown globular material. The neoplastic cells were fusiform to spindloid, formed interlacing bundles, and had moderate amounts of darkly eosinophilic cytoplasm. The nuclei were ovoid and had a stippled chromatin pattern with numerous, variably sized prominent nucleoli. There was marked anisokaryosis and anisocytosis. Binucleated and multinucleated cells as well as karyomegaly were multifocally observed. The mitotic count was 20 in 10 high powered fields (HPF, FN22), and there were occasional bizarre mitotic figures (Figure 2).

**Figure 3:**
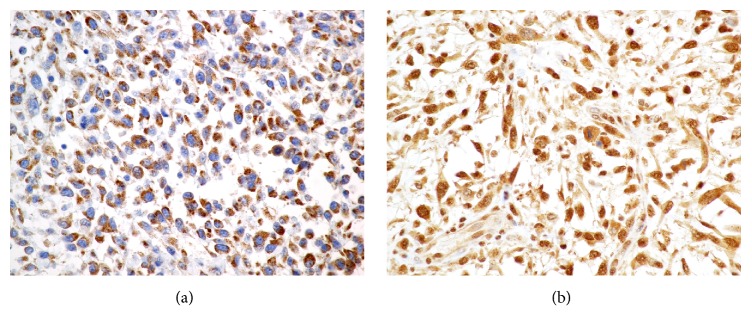
Positive immunohistochemical staining of the tumor for (a) VEGFr and (b) PDGFr.

A grade 3 soft tissue sarcoma was diagnosed based on the degree of necrosis, cellular atypia, and the high mitotic count. The neoplasm had narrow but completely excised surgical margins. The surgical wound healed without any complications; however, multiple subcutaneous nodules were identified at the excision site ten weeks later. Repeat fine needle aspiration cytology confirmed a recurrence of the sarcoma. Hematology, serum biochemistry, and urinalysis were unremarkable, and three-view thoracic radiographs did not identify metastatic disease to the lungs. A second, broad excision of the injection site with 3 cm margins was performed. The neoplasm appeared histologically similar to the previously excised grade 3 soft tissue sarcoma with an increased mitotic count of 30/10 HPF. The sarcoma had focally infiltrated the skeletal muscle. Excision was reported to be complete with narrow margins. Neoplastic cells were immunohistochemically positive for Vascular Endothelial Growth Factor receptor (VEGFr) (Figure 3(a)), Platelet Derived Growth Factor receptor (PDGRr) (Figure 3(b)), Stem Cell Factor (SCF), and Epithelial Growth Factor Receptor (EGFR) and negative for VEGF, PDGR, KIT, and p-53.

Based on the expression of VEGFr and PDGFr, a response to targeted tyrosine kinase inhibitor therapy with toceranib, a small molecule inhibitor of VEGFr2 and PDGFr*β*, was hypothesized. Toceranib was initially administered orally at a dose of 2.1 mg/kg and then increased to 2.8 mg/kg on a Monday-Wednesday-Friday schedule. No adverse effects were noted at these doses and no hypertension or proteinuria was detected. Repeat CBC and serum biochemistry profiles were normal throughout the course of therapy and thoracic radiographs did not show any evidence of metastatic disease. A small cluster of subcutaneous nodules was identified at the excision site fifty weeks after the first surgery and a third complete excision with 3 cm margins was performed. Histology confirmed a recurrence of the grade 3 soft tissue sarcoma and the toceranib was continued. At the time of submission of this manuscript, the dog remains in remission ninety-three weeks after initial diagnosis.

## 3. Discussion

Injection-site sarcomas have been rarely reported in dogs. In a pathological study, soft tissue sarcomas in fifteen dogs that developed at sites where previous vaccinations may have been given were compared with canine soft tissue sarcomas that developed at nonvaccination sites, as well as with injection-site sarcomas from cats. A histologic pattern consistent with that of feline injection-site sarcomas [9] was reported in these dogs [10]. In this same study, aluminum deposits attributed to vaccine adjuvants were found in half of the canine vaccination associated sarcomas but were in none of the sarcomas from nonvaccination sites. Based on the characteristic morphology and the detection of aluminum which linked the tumors to the vaccinations, the authors concluded that injection-site sarcomas may develop in dogs [10]. No follow-up information or response to treatments was provided. The clinical presentation and the morphologic phenotype of the sarcoma reported here are most consistent with a diagnosis of an injection-site sarcoma.

The association of feline soft tissue sarcomas with rabies and feline leukemia virus vaccinations was made over twenty years ago [11], and subsequent epidemiological studies have estimated an incidence of 0.3 to 1.0 injection-site sarcomas per 10,000 vaccinations in the US and Europe [8]. The mechanism of tumorigenesis of injection-site sarcomas is incompletely understood, but chronic inflammation at the injection site and potential genetic predisposition are believed to lead to malignant transformation, especially suppression of tumor suppressor genes such as p53 [12–15]. There was no evidence of expression of p53, as commonly observed in neoplasms with p53 mutations, in the case presented here.

Sarcomas in cats have been reported to develop as early as 4 months and up to several years after vaccination, although in many instances the prepatent period is unknown [8]. Feline injection-site sarcomas are highly invasive with a propensity for local recurrence and investigation about prognostic factors is ongoing [16–18]. Advanced imaging, such as computed tomography (CT) or magnetic resonance imaging (MRI), has been advocated for presurgical planning. Treatment options include surgery, radiotherapy, and chemotherapy. Aggressive surgical resection with margins of up to 5 cm or amputation, if indicated, is oftentimes necessary to control local spread. Distant metastasis, especially to the lungs, occurs in up to 21% of affected cats [17]. While there was no evidence of metastatic disease throughout the course of disease in this dog, the neoplasm did locally recur ten weeks and fifty weeks after complete surgical excision.

Toceranib phosphate is an oral, small molecule split receptor tyrosine kinase (RTK) inhibitor that is licensed for use in the US and Europe at the maximally tolerated label dose of 3.25 mg/kg for high-grade mast cell tumors in dogs. Toceranib possesses antiangiogenesis and antitumor activity due to its potent inhibition of targets, including VEGFr, PDGFr, and KIT [19]. Biologic activity has been demonstrated for a variety of tumors in dogs, including carcinomas, sarcomas, and hematopoietic neoplasms. Partial responses and stable disease are more commonly observed than complete responses [20]. Nevertheless, toceranib has become a valuable extra label treatment option as an adjunct to the definitive surgical removal of certain tumors, or as a primary therapy for select, nonresectable neoplasms. Expression of tyrosine kinase receptors that are targeted by toceranib such as PDGFr has been documented in canine osteosarcomas [21]. Apoptosis of canine osteosarcoma cells has been induced in vitro using another tyrosine kinase inhibitor, masitinib mesylate, which targets KIT and PDGFr [22]. In one study, the expression of VEGF and VEGFr was found to be 92% and 100%, respectively, on 24 spontaneously occurring canine cutaneous fibrosarcomas and the expression of both correlated with histologic grade [23]. In another study of 40 canine soft tissue sarcomas, the expression of VEGFr and PDGFr was detected by IHC in 67.5% and 80% of examined neoplasms, respectively [24], suggesting that these receptors in soft tissue sarcomas may be potential targets for toceranib therapy. While expression of PDGF, EGF and its receptors, and Transforming Growth Factor (TGFß) has been reported in feline injection-site sarcomas [14], a recent report of 18 cats with unresectable injection-site sarcomas that were treated with toceranib failed to show any clinical responses [25]. Prolonged survival was observed in one dog with an injection-site sarcoma treated with surgical excision, carboplatin, toceranib, and cyclophosphamide [26].

In the dog in this report, the expression of VEGFr, PDGFr, EGFr, and SCF was demonstrated by immunohistochemistry. Toceranib, since it targets VGEFr and PDGFr, was selected with the intent of reducing the potential for local regrowth and distant metastasis [19]. The dose administered, ranging from 2.1 to 2.8 mg/kg, was chosen empirically based on recent evidence that lower doses are associated with fewer gastrointestinal side effects [27]. This same study reported stable disease for 30 days in 5 out of 7 dogs with spontaneously occurring soft tissue sarcomas treated with toceranib dosed at 2.4–2.9 mg/kg every other day [27]. The dog in this case report has experienced a prolonged disease-free interval while receiving toceranib with no adverse effects noted. The favorable clinical response may be attributable to the toceranib in conjunction with wide surgical resection.

In conclusion, the association of vaccinations with the occurrence of a high-grade soft tissue sarcoma several weeks after the injections in the dog in this report and the morphologic phenotype of the reported sarcoma is most consistent with a diagnosis of an injection-site sarcoma. This association does not constitute definitive proof that the vaccinations caused the sarcoma. Expression of tyrosine kinase receptors was investigated by immunohistochemistry, and a targeted therapy was elected based on VEGFr and PDGFr expression. Aggressive surgical resection of the soft tissue sarcoma and treatment with toceranib appeared to be useful in maintaining a sustained remission.
